# Does universal insurance influence disparities in high-quality hospital use for inpatient pediatric congenital heart defect care within the first year of diagnosis?

**DOI:** 10.1186/s12913-023-09668-1

**Published:** 2023-06-28

**Authors:** Amber El-Amin, Tracey Koehlmoos, Dahai Yue, Jie Chen, Peyman Benharash, Luisa Franzini

**Affiliations:** 1 Center for Health Services Research, Uniformed Services University of Health Sciences, Bethesda, MD US; 2 Department of Health Policy and Management, School of Public Health, University of Maryland, College Park, MD US; 3 Cardiovascular Outcomes Research Laboratories (CORELAB), University of California, Los Angeles, CA US; 4 Department of Surgery, University of California, Los Angeles, CA US

**Keywords:** The Military Health System Data Repository, Congenital heart disease, Socioeconomic status, Military personnel, Universal healthcare system, Minorities

## Abstract

**Background:**

Healthcare disparities are an issue in the management of Congenital Heart Defects (CHD) in children. Although universal insurance may mitigate racial or socioeconomic status (SES) disparities in CHD care, prior studies have not examined these effects in the use of High-Quality Hospitals (HQH) for inpatient pediatric CHD care in the Military Healthcare System (MHS). To assess for racial and SES disparities in inpatient pediatric CHD care that may persist despite universal insurance coverage, we performed a cross-sectional study of the HQH use for children treated for CHD in the TRICARE system, a universal healthcare system for the U.S. Department of Defense. In the present work we evaluated for the presence of disparities, like those seen in the civilian U.S. healthcare system, among military ranks (SES surrogate) and races and ethnicities in HQH use for pediatric inpatient admissions for CHD care within a universal healthcare system (MHS).

**Methods:**

We conducted a cross-sectional study using claims data from the U.S. MHS Data Repository from 2016 to 2020. We identified 11,748 beneficiaries aged 0 to 17 years who had an inpatient admission for CHD care from 2016 to 2020. The outcome variable was a dichotomous indicator for HQH utilization. In the sample, 42 hospitals were designated as HQH. Of the population, 82.9% did not use an HQH at any point for CHD care and 17.1% used an HQH at some point for CHD care. The primary predictor variables were race and sponsor rank. Military rank has been used as an indicator of SES status. Patient demographic information at the time of index admission post initial CHD diagnosis (age, gender, sponsor marital status, insurance type, sponsor service branch, proximity to HQH based on patient zip code centroid, and provider region) and clinical information (complexity of CHD, common comorbid conditions, genetic syndromes, and prematurity) were used as covariates in multivariable logistic regression analysis.

**Results:**

After controlling for demographic and clinical factors including age, gender, sponsor marital status, insurance type, sponsor service branch, proximity to HQH based on patient zip code centroid, provider region, complexity of CHD, common comorbid conditions, genetic syndromes, and prematurity, we did not find disparities in HQH use for inpatient pediatric CHD care based upon military rank. After controlling for demographic and clinical factors, lower SES (Other rank) was less likely to use an HQH for inpatient pediatric CHD care; OR of 0.47 (95% CI of 0.31 to 0.73).

**Conclusions:**

We found that for inpatient pediatric CHD care in the universally insured TRICARE system, historically reported racial disparities in care were mitigated, suggesting that this population benefitted from expanded access to care. Despite universal coverage, SES disparities persisted in the civilian care setting, suggesting that universal insurance alone cannot sufficiently address differences in SES disparities in CHD care. Future studies are needed to address the pervasiveness of SES disparities and potential interventions to mitigate these disparities such as a more comprehensive patient travel program.

**Supplementary Information:**

The online version contains supplementary material available at 10.1186/s12913-023-09668-1.

## Background

Congenital heart disease (CHD) has an incidence of 0.8% to 1.2% worldwide, making it the most common birth defect [1]. CHD is the leading cause of deaths related to birth defects, accounting for ~ 40% of deaths in children with birth defects [2]. Although major advances in the diagnosis and longitudinal treatment of CHD have yielded significant improvements in mortality, race based disparities persist [3]. Race, ethnicity, socioeconomic status (SES), and residential location have shown to be associated with mortality in children who underwent surgery for CHD [4]. Racial minorities, such as Black and Hispanic populations, have been demonstrated to face increased mortality and resource use during CHD-related hospitalizations [1]. Military rank has been correlated with SES with enlisted personnel having lower SES and officers having higher SES [5]. Whether such disparities stem from patient related factors or related to the quality of treatment facilities have been more recently questioned.

Data show that patients with CHDs who are Black or have public insurance are more likely to obtain medical care at facilities with worse patient outcomes [6, 7]. Hospital operative volume and teaching designation have been utilized as a surrogate for quality in CHD care. It was noted that critically ill CHD patients at low-volume/non-teaching facilities faced higher odds of inpatient mortality when compared to CHD patients at high-volume/teaching hospitals [8]. Black patients have demonstrated to be more likely to receive surgical care from lower volume/higher mortality surgeons [9]. Furthermore, a Center of Excellence (CoE) designation has been recommended as a manner to create a network of high-quality care and to affect hospital and provider selection for patients. Previous studies of CoE programs have been limited to surgical procedures and have had mixed results [10]. These inconsistencies may result from how hospital quality of care is measured, which variables are controlled, or how outcomes are measured. High-quality hospital (HQH) are hospitals that provide effective and safe care with a culture of excellence, resulting in optimal health [11]. There have been no studies using U.S. Military Health System (MHS) data to look for possible disparities based upon race and ethnicity or SES for inpatient pediatric CHD care.

In the present work we evaluated for the presence of disparities, like those seen in the civilian U.S. healthcare system, among military ranks (SES surrogate) and races and ethnicities in HQH use for pediatric inpatient admissions for CHD care within a universal healthcare insurance system. We hypothesized White race to be independently associated with higher likelihood of receiving care at an HQH. We hypothesized Officer rank to be independently associated with higher likelihood of receiving care at an HQH.

## Methods

### Study design

We conducted a retrospective cross-sectional study with U.S. data from 2016 to 2020.

### Study population

Since there is a particular interest in what facility an initial corrective surgery takes place, this study included all pediatric inpatient CHD care received within the first year post-CHD diagnosis in U.S. hospitals from 2016 to 2020. TRICARE beneficiaries with a diagnosis of CHD were tabulated based on relevant ICD-10 (International Classification of Diseases, 10^th^revision) codes as reported previously [12]. The study population was categorized into four groups: single ventricle CHD, moderate-complex CHD, simple CHD, or other CHD based on the hierarchy of the complexity of CHD [12] (Additional file 1). Records with other supplemental insurance as well as those lacking at least one year of follow-up care or treatment at a military facility outside of the U.S. were excluded. Furthermore, children with patent ductus arteriosus and/or isolated congenital anomaly of the peripheral, or cerebral vascular system [12], or cardiac transplantation were excluded to enhance cohort homogeneity. Finally, patients were excluded if they were missing data on variables of interest: race or rank (approximately *N* = 883 or 7.69% were missing race). Of the 7.69% missing race, there were 6.98% (*N* = 820) missing marital status.

### Data source

We used the U.S. MHS Data Repository (MDR) to identify MHS beneficiaries ages 0 to 17 years who received CHD care from 2016 to 2020. The MDR provides information at the patient encounter level, including diagnoses, treatments, patient demographic characteristics, and the facility in which care was delivered. The MDR includes data for beneficiaries who use U.S. MHS facilities which are operated by the Department of Defense (DoD), also known as direct care for military treatment, and for beneficiaries who receive care in the civilian private sector that is funded by TRICARE, also known as purchased care. TRICARE tracks claims data separately for purchased and direct care and includes several component insurance plans but is not responsible for care within military combat zones or care received at Veterans Affairs facilities [13]. The dataset has unique hospital identification numbers, which permits facility comparisons. The TRICARE program covers 9.6 million beneficiaries including approximately 20% active military personnel and a combination of dependents and retirees, serving as a sociodemographic representation of the U.S. population younger than age 65 [13]. This protocol was approved by the Institutional Review Board at the University of Maryland, College Park (Approval Number: 1576246–2).

### Study outcome

The dependent variable, HQH use, was a dichotomous indicator for HQH utilization categorized as (1) yes or (0) no. This study categorized HQH based on The Society of Thoracic Surgeons (STS) Congenital Heart Surgery Database (CHSD), which is the largest congenital and pediatric cardiac surgical clinical data registry in the world and includes the analysis of outcomes and the improvement of quality for congenital heart surgeries [14]. The STS CHSD covers hospitals’ results over a four-year period and presents respective observed-to-expected (O/E) mortality ratios for operative mortalities, which incorporates a risk adjustment to account for differences in case mix, serving as a platform for benchmarking performance and improving quality for CHD care [15]. Hospitals with an overall O/E mortality ratio of equal to or less than one were designated as HQH, since these hospitals had fewer deaths than expected based on the case mix treated at the hospital [16]. Hospitals with an O/E mortality ratio greater than one were categorized as non-HQH. The STS CHSD data from the 2015 to 2018 period was used to classify hospitals, totaling 42 HQH within the U.S. In addition, 984 hospitals that did not report to the STS for CHD care were categorized as non-HQH.

### Independent Variables

The primary independent variable was self-declared race (White, Black, or Other race to include Asian, Pacific Islander, Native American or Other) by the military member (also known as the sponsor). Sponsor race was used as an imputation of dependent (i.e. child) race which is a tested method of imputation for this population [17]. The secondary independent variable measures disparities in HQH use based on sponsor rank, as a proxy for SES, of Junior Enlisted (E1-E4), Senior Enlisted (E5-E9), Officer, or Other ranks (cadets, midshipmen, officer candidates, reserve officer training corps members, and warrant officers).

Covariates were defined at the time of index admission post initial CHD diagnosis. The following model covariates were included: age (1 to 30 days, 31 days to less than 1 year [reference], 1 year to 17 years), gender (male and female [reference]) [18], and sponsor marital status (currently married, not married to include divorced and widowed [reference]) [19]. Type of TRICARE insurance (Prime (managed care at a military treatment facility care for active duty, retired, activated guard/reserve, survivors, Medal of Honor recipients and their families) [reference], Other (self-managed preferred provider plan that does not require care at a military treatment facility), Unenrolled), sponsor service branch (Army [reference], Air Force, Marine Corps, Navy, Other service)[17], and proximity to HQH based on patient zip code centroid and HQH zip code centroid within 100-mile radius (yes or no [reference]) [20], and provider region (South [reference], West, Midwest, Northeast). Complexity of CHD (simple CHD [reference], moderate-complex CHD, single ventricle CHD, other CHD), common comorbid conditions (yes or no [reference]), genetic syndromes (Down’s, Noonan’s, DiGeorge, Holt-Oram, Turner, and Williams-(Beuren); yes or no [reference]) [12], and prematurity (gestational age less than 37 weeks; yes or no [reference]). Patients with multiple CHD diagnoses were assigned to the CHD complexity category associated with the most complex diagnosis. A list of ICD-10 codes for included CHD diagnoses, genetic syndromes, and common comorbid conditions may be found in Additional file 1.

### Statistical analysis

Patient demographic and medical characteristics and medical center factors were characterized using descriptive statistics and between-group (HQH use vs non-HQH use) differences measured by chi-square. The relationship between HQH use and each category of sponsor rank and sponsor race were modeled using a multivariable logistic regression to estimate the probability of HQH use, while adjusting for patient and hospital characteristics, calendar year fixed effects, and region as fixed effects. Elastic Net regularization was used for covariate selection assistance, which selects variables to improve out-of-sample generalizability [21]. The adjusted odds ratios (OR), *p*-values, along with 95% confidence intervals (CI) were reported. All analyses were performed using Stata statistical software (Version 17.0 Statacorp, College Station, Texas). Two-sided *p*-values less than 0.05 were considered statistically significant.

## Results

**Fig. 1:**
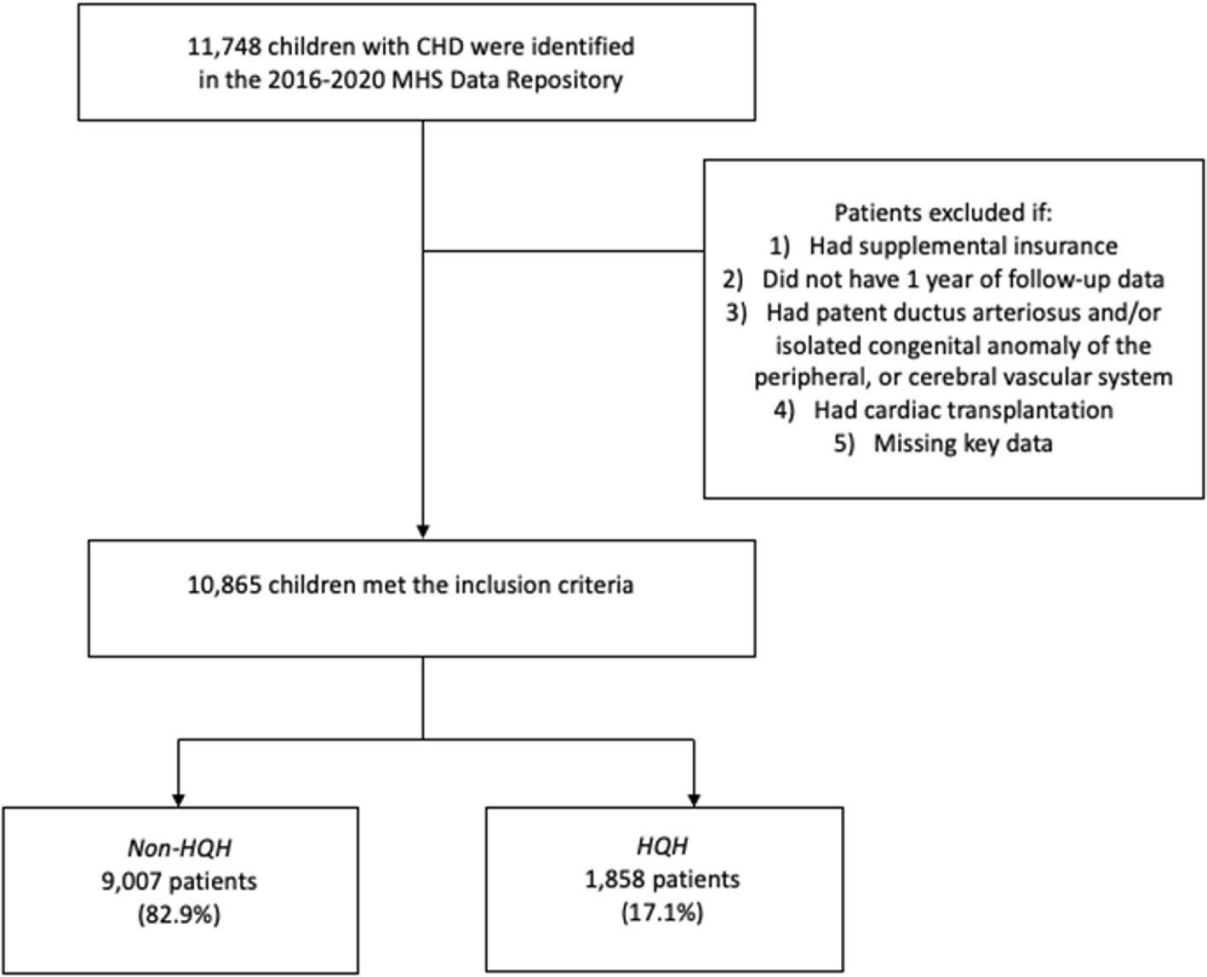
Flowchart of Study Population

During the study period (2016–2020), we identified 11,748 children with CHD. After exclusion criteria were applied (7.69% missing race), there were 10,865 persons that met the inclusion criteria. In the sample, 42 hospitals were designated as HQH. Of the population, 82.9% did not use an HQH at any point for inpatient pediatric CHD care and 17.1% used an HQH at some point for inpatient CHD care (Fig. 1). Furthermore, 73% were White, 17.1% were Black, and 9.9% were Other races. Among White race, 83% used a non-HQH and 17% used an HQH. Among Black race, 83.7% used a non-HQH and 16.3% used an HQH. Among Other race, 80.5% used a non-HQH and 19.5% used an HQH. Junior Enlisted, Senior Enlisted, Officer, and Other rank HQH use represented 16.3%, 17.6%, 18.6%, and 10.6%, respectively. Of the sample, Junior Enlisted members represented 28.2%, Senior Enlisted represented 50.5%, Officers represented 17.6%, and Other members represented 3.7%. Among Junior Enlisted, 83.7% used a non-HQH and 16.3% used an HQH. Among Senior Enlisted, 82.4% used a non-HQH and 17.6% used an HQH. Among Officers, 81.4% used a non-HQH and 18.6% used an HQH. Among Other rank, 89.4% used a non-HQH and 10.6% used an HQH. Among patients who lived within 100 miles of an HQH, 69.6% used a non-HQH and 30.4% used an HQH.

Among those who used an HQH versus a non-HQH, 72.5% were White race compared to Black and Other races (16.2%, and 11.3%, respectively), while 26.8% were Junior Enlisted, 51.8% were Senior Enlisted, 19.1% were Officers, and 2.3% were Other rank. The admission characteristics and a comparison of these characteristics between HQH and non-HQH use are listed for the entire cohort in Table 1. Among those who used HQH, more patients were 1 to 5 years old, had Prime insurance coverage, were affiliated with the Marine Corps or Navy, lived with 100 miles of an HQH, lived in the Northeast, had a single ventricle CHD, and had a genetic syndrome or comorbid condition.

**Table 1:** Characteristics Associated with HQH Use, 2016–2020

	Total = 10,865	Non-HQH = 9,004		HQH = 1,861		*P*-value
	N	N	(%)	N	(%)	
Race						0.07
White	7,931	6,582	83.0	1,349	17.0	
Black	1,857	1,555	83.7	302	16.3	
Other race	1,077	867	80.5	210	19.5	
Sponsor Pay Grade						< 0.01*
Jr. Enlisted	3,067	2,568	83.7	499	16.3	
Sr. Enlisted	5,491	4,527	82.4	964	17.6	
Officer	1,909	1,553	81.4	356	18.6	
Other Rank	398	356	89.4	42	10.6	
Age Group						< 0.01*
31 to 364 days	3,247	2,574	79.3	673	20.7	
0 to 30 days	6,564	5,699	86.8	865	13.2	
1 to 5 years	458	312	68.1	146	31.9	
6 to 17 years	596	419	70.3	177	29.7	
Gender						0.05*
Female	5,015	4,195	83.6	820	16.4	
Male	5,850	4,809	82.2	1,041	17.8	
Married						0.60
No	873	729	83.5	144	16.5	
Yes	9,992	8,275	82.8	1,717	17.2	
Insurance						< 0.01*
Prime	4,283	3,439	80.3	844	19.7	
Other Insurance Type	6,270	5,289	84.4	981	15.6	
Unenrolled	312	276	88.5	36	11.5	
Sponsor Service						< 0.01*
Army	4,959	4,186	84.4	773	15.6	
Air Force	2,690	2,331	86.7	359	13.3	
Marine Corps	1,032	799	77.4	233	22.6	
Navy	1,866	1,421	76.2	445	23.8	
Other Service	318	267	84.0	51	16.0	
Proximity within 100 Miles of an HQH						< 0.01*
No	5,987	5,609	93.7	378	6.3	
Yes	4,878	3,395	69.6	1,483	30.4	
Provider Region						
South	5,732	4,945	86.3	787	13.7	< 0.01*
West	3,108	2,440	78.5	668	21.5	
Midwest	1,423	1,156	81.2	267	18.8	
Northeast	602	463	76.9	139	23.1	
CHD Complexity						< 0.01*
Simple CHD	6,890	6,005	87.2	885	12.8	
Moderate-Complex CHD	2,284	1,638	71.7	646	28.3	
Single Ventricle CHD	244	130	53.3	114	46.7	
Other CHD	1,447	1,231	85.1	216	14.9	
Genetic Syndrome						< 0.01*
No	10,769	8,943	83	1,826	17	
Yes	96	61	63.5	35	36.5	
Comorbid Condition						< 0.01*
No	9,649	8,079	83.7	1,570	16.3	
Yes	1,216	925	76.1	291	23.9	
Prematurity						< 0.01*
No	7,341	5,979	81.4	1,362	18.6	
Yes	3,524	3,025	85.8	499	14.2	

^*^Denotes significance at the .05 level

Other race defined as Asian or Pacific Islander, American Indian or Alaskan native

Other Rank defined as Other Rank defined as cadets, midshipmen, officer candidates, reserve officer training corps members, warrant officers

Other Insurance type defined as Extra, Standard, Select, and Young Adult

Controlling for demographic variables and risk factors, odds ratios, confidence intervals, and *p*-values are depicted in Table 2. There was no statistically significant association between race and HQH use. Compared to Junior Enlisted, Other rank was statistically less likely to use HQH; OR of 0.47 (95% CI of 0.31 to 0.73). Compared to patient ages 31 days to 364 days, patient ages 0 to 30 days old were statistically less likely to use HQH; OR of 0.53 (95% CI of 0.41 to 0.68). Compared to patient ages 31 days to 364 days, patient ages 1 to 5 years old were statistically more likely to use HQH; OR of 1.32 (CI of 1.01 to 1.71). Premature patients were statistically less likely to use HQH; OR 0.72 (CI of 0.58 to 0.88). Patient residence proximity within 100 miles of an HQH was statistically more likely to use HQH; OR 6.77 (95% CI of 4.63 to 9.90). Compared to simple CHD diagnoses, moderate-complex CHD diagnoses and single ventricle CHD diagnoses were statistically more likely to use HQHs; ORs of 2.72 (95% CI of 2.21 to 3.34) and 6.51 (95% CI of 4.22 to 10.04), respectively. Patients with a genetic syndrome were statically more likely to us HQH; OR of 1.90 (95% CI of 1.24 to 2.92).

**Table 2:** Results of Regression for HQH Use, 2016–2020

Characteristics	Multivariable OR	*P*-Value	95% Conf. Interval
Race			
White [ref]			
Black	0.93	0.40	(0.78, 1.10)
Other Race	1.09	0.44	(0.88, 1.35)
Sponsor Pay Grade			
Jr. Enlisted [ref]			
Sr. Enlisted	0.88	0.06	(0.77, 1.00)
Officer	0.93	0.58	(0.73, 1.19)
Other Rank	0.47	< 0.01*	(0.31, 0.73)
Patient Age Group			
31 to 364 days [ref]			
0 to 30 days	0.53	< 0.01*	(0.41, 0.68)
1 to 5 years	1.32	0.04*	(1.01, 1.71)
6 to 17 years	1.16	0.28	(0.89, 1.52)
Gender			
Female [ref]			
Male	1.09	0.11	(0.98, 1.21)
Married			
No [ref]			
Yes	1.07	0.53	(0.86, 1.34)
Insurance			
Prime [ref]			
Other Insurance Type	0.84	0.08	(0.69, 1.02)
Unenrolled	0.69	0.05	(0.47, 1.01)
Sponsor Service			
Army [ref]			
Air Force	0.89	0.44	(0.67, 1.19)
Marine Corps	1.40	0.29	(0.75, 2.59)
Navy	1.15	0.62	(0.66, 2.00)
Other	0.83	0.54	(0.45, 1.52)
Proximity within 100 Miles of an HQH			
No [ref]			
Yes	6.77	< 0.01*	(4.63, 9.90)
Provider Region			
South [ref]			
West	1.59	0.32	(0.63, 3.98)
Midwest	1.62	0.24	(0.72, 3.61)
Northeast	1.16	0.75	(0.47, 2.84)
CHD Complexity			
Simple CHD [ref]			
Moderate-Complex CHD	2.72	< 0.01*	(2.21, 3.34)
Single Ventricle CHD	6.51	< 0.01*	(4.22, 10.04)
Other CHD	1.11	0.29	(0.91, 1.35)
Genetic Syndrome			
No [ref]			
Yes	1.90	< 0.01*	(1.24, 2.92)
Comorbid Condition			
No [ref]			
Yes	1.17	0.13	(0.95, 1.44)
Prematurity			
No [ref]			
Yes	0.72	< 0.01*	(0.58, 0.88)

^*^Denotes significance at the .05 level

Other Race defined as Asian or Pacific Islander, American Indian or Alaskan native

Other Rank defined as cadets, midshipmen, officer candidates, reserve officer training corps members, warrant officers

Other Insurance type defined as Extra, Standard, Select, and Young Adult

## Discussion

This study evaluated for disparities among military ranks (SES) and race in a universal healthcare system for inpatient CHD care. We did not find disparities in HQH use for inpatient pediatric CHD care based on race. We did find disparities in HQH use for inpatient pediatric CHD care based upon military rank. Retrospective analysis was conducted of all inpatient admissions for CHD care between years 2016 to 2020. Of the population, 82.9% did not use an HQH at any point for pediatric inpatient CHD care and 17.1% used an HQH at some point for pediatric inpatient CHD care. Similar to previously published work, compared to low volume surgery centers, high volume surgery centers represented 82% of CHD hospitalizations [22]. Military rank as a SES surrogate did appear to have a statistically significant impact on HQH use. This effect was distinct even after controlling for comorbidities, risk factors, and demographics. Other ranks (lower SES) were less likely to use an HQH for inpatient pediatric CHD care; OR of 0.47 (95% CI of 0.31 to 0.73). The largest statistically significant association was the likelihood that a person who lived within 100 miles of an HQH used an HQH. The second largest association was the likelihood that a person used an HQH who had a single ventricle CHD.

Disparities related to race/ethnicity in the care provided in the civilian health care system are common. It has been demonstrated that Hispanics have an increased likelihood of complications post CHD surgery [23], while non-Hispanic Blacks are more at risk for mortality post CHD surgery when compared to non-Hispanic Whites [2]. We did not find disparities in HQH use for inpatient pediatric CHD care based on race, which may be due to the MHS unique universal insurance coverage. Since lack of or inadequate health insurance coverage is one of the most impactful social determinants of health and the largest barrier to health care access [24], it is likely that universal insurance and equitable access to care have the prospective to profoundly reduce or eliminate racial disparities for pediatric CHD care. Similarly, it has been shown that racial disparities in use of inpatient resources for CHD care are decreased when the mother has higher levels of education and private insurance [1].

Previously published work demonstrated associations between lower SES and worse outcomes after CHD surgery for pediatric patients. There is a strong relationship between SES and increased rates of complications from CHD [1]. Our study found that SES was associated with HQH use. Other rank (lower SES) was less likely to use an HQH for pediatric inpatient CHD care. Other rank consisted of cadets, midshipmen, officer candidates, reserve officer training corps members, and warrant officers. Other rank HQH use may be lower because members in training or college may be less likely to be able to leave their training for prolonged periods of time. In addition, military members in training status (Other ranks) are less likely to have the income to fund patient travel or accommodations when these expenses are not covered by insurance. For this reason, Other ranks may be less likely to travel long distances to access care. A majority of beneficiaries (81%) whose sponsor was of Other rank were 0 to 30 days old at the time of initial admission. Compared to patient ages 31 days to 364 days, patient ages 0 to 30 days old were statistically less likely to use HQH. Age was controlled in our study and does not explain all the observed disparities but may account for some of these disparities. All beneficiaries in a universal healthcare system have the same coverage, reducing the potential impact of different levels of insurance coverage and providers [5, 25]. For this reason, access to care should be comparatively similar for all military rank groups (SES groups) [5]. However, our study still found significant differences among Junior Enlisted rank and Other rank. These disparities should have been minimized in a universal healthcare system. Although the reason for the negative association between Other rank and HQH use, compared to Junior Enlisted, are not clear, more efforts should be made to increase awareness of HQH. There should be a more dedicated focus to increase awareness of HQH to lower SES groups.

This study noted the largest association with HQH use as proximity to HQH, which may be due to referral patterns or patient preference. Since the geographic distribution of HQH leaves many areas across the U.S. without easy access to a pediatric CHD HQH, referral patterns and patient preference may be affected by convenience, travel costs, or lodging costs. For this reason, patients who require CHD care would benefit from comprehensive insurance coverage that covers travel and accommodation costs. This study noted the second largest association with HQH use to be with patients who had a single ventricle CHD (most severe CHD). Similarly, previously published work in this field showed that high-volume hospitals performed a large proportion of higher complexity procedures [26]. This finding may be due to the greater level of skill and clinical experience required to address higher severity of CHD. This is the first study to characterize HQH use for inpatient pediatric CHD care by race and military rank for MHS beneficiaries.

It is likely that other variables existing prior to pediatric inpatient CHD care may play a role in observed disparities in HQH use that are correlated with military rank. These include a person’s training status and ability to access an appropriate level of care. We propose that these characteristics of a population (SES group) play a role in the variances seen among SES groups.

### Policy Implications

We did not find disparities in HQH use for inpatient pediatric CHD care based on race. Poverty, access to care, and insurance status have demonstrated to effect outcomes of CHD surgery [27]. Since lack of or inadequate health insurance coverage is one of the most impactful social determinants of health and the largest barriers to health care access [24], it is likely that universal insurance and equitable access to care have the prospective to profoundly reduce or eliminate racial disparities for pediatric CHD care. Although race and ethnicity are critical in understanding disparities in HQH use for pediatric inpatient CHD, understanding SES factors within racial/ethnic groups that may contribute to non-HQH use is imperative to pinpoint targets for intervention. We did not find disparities in HQH use between Junior Enlisted and Senior Enlisted ranks nor did we find disparities in HQH use between Junior Enlisted and Officer ranks. However, we did find disparities in HQH use for Other ranks. Our analysis showed that Other ranks were associated with lower odds of HQH use than Junior Enlisted rank. The presence of disparities in the care received in the civilian health care system suggests that providing universal insurance alone will not fully combat differences in healthcare utilization or patient care for patients with differing SES. In this setting, more comprehensive insurance coverage of patient travel and accommodations may minimize SES related disparities.

Despite access to performance metrics on quality of care to include mortality, 82.3% of the sample never used a HQH at any point for CHD care. Similar to previously published work, compared to low volume surgery centers, high volume surgery centers represented 82% of CHD hospitalizations [22]. It is likely that non-HQH continue to provide CHD care to the military beneficiary population because of institutional differences in awareness of quality metrics (such as the STS CHSD), perceived lack of access, and barriers to access (such as referral requirements and inability to fund patient travel and accommodations).

The findings suggest that a universal healthcare system, such as the MHS, has the potential to minimize disparities but not eliminate disparities. Given the findings of this study, it is vital for clinicians and patient advocacy groups to urge policymakers to target implementation of transparent reporting of CHD surgery outcomes by medical center, which can be used to stratify surgery centers based on CHD complexity [28]. These efforts will not only help to identify standardized quality metrics and best practices but can also be used to further regionalization and mandatory reporting efforts. In addition, it is important to determine referral patterns and access to HQH [28]. The aforementioned efforts can be used to steer patients to proven HQH to improve care for CHD patients and reduce barriers to utilize HQH. In fact, standardized quality metrics should be used to determine the in-network facilities for the MHS, including only those facilities with proven high quality of care. Due to the greater level of skill and clinical experience required to address higher severity of CHD, policymakers should direct patients to HQH for more severe CHDs.

### Limitations

This study’s results should be considered with several limitations. Since 84.7% of hospitals participate in public reporting via STS, but not all U.S. centers participate, inability to fully classify hospitals as HQH versus non-HQH may be a limitation to this study. Although we used hospital quality rating for congenital cardiac surgery as reported by the STS, the contributing data span a 4-year period and may not reflect real time changes in center performance. Additionally, TRICARE claims data are consistently audited for accuracy, reliability, and completeness; however, not all centers submit complete data for all data elements thus are not included in this analysis. Next, CHD operations were grouped within complexity of CHD diagnosis categories, so it may not be appropriate to apply the results to all operations within a group. Race data was only available for the active duty military parent, so when maternal race was unknown infant race was imputed based on paternal race [17]. Approximately 7.69% of the dataset was missing sponsor race data and these observations were dropped from the study which may cause bias in estimates. However, multiple imputation was tested to impute missing values of race, resulting in almost the same coefficients as non-imputed data. Imputation based on geocoding and surnames have proven superior for predicting race [29], but this dataset did not include data on an individual’s specific address nor their surname. For this reason, we did not further impute the dataset. Since this study focused on inpatient care within the first year post initial CHD diagnosis, this study’s findings may not reflect the full range of health system utilization. It is important to note that the Other rank represented an extremely small percent of the study population (3.7%), which could create bias in the estimates. Despite these limitations, this analysis contributes to the literature on CHD care by providing the first estimates of the characteristics associated with HQH use for inpatient pediatric CHD care in the U.S.

## Conclusions

The current study demonstrates that only 17.1% of pediatric CHD patients were treated at HQH at some point for related care. Our study found that a multitude of factors contribute to HQH utilization for a patient who requires pediatric CHD care. This may suggest that education about the importance of care in a HQH is needed at the point of initial CHD diagnosis, particularly for those with more complex CHD diagnoses. Although this study did not find racial disparities in HQH use, we noted disparities in HQH utilization based on SES (military rank) which are likely exacerbated by social risks. The findings suggest that a universal healthcare insurance system, such as the MHS, has the potential to minimize disparities but not eliminate disparities. A focus on standardized quality metrics that clearly designate a facility as a HQH are needed to broadly reduce selection bias. The abovementioned efforts can be used to steer patients to proven HQH to improve care for CHD patients. Furthermore, a more comprehensive travel benefit in the MHS, to cover CHD related patient travel and accommodations associated with HQH use, may minimize SES related disparities. These results are generalizable to single-payer systems and provide detail on how racial disparities can be mitigated with universal insurance coverage. Further focus on which factors may be contributing to non-HQH use, especially a more comprehensive travel benefit, is necessary and will provide critical information for devising interventions.

## Supplementary Information

**Additional file 1.**

## Data Availability

The data that support the findings of this study are available from the Defense Health Agency, but restrictions apply to the availability of these data, which were used under license for the current study, and so are not publicly available. The data is only available to the principal investigator, Amber El-Amin.

## Abbreviations

CoE: Center of Excellence
CI: Confidence Intervals
CHD: Congenital Heart Disease
CHSD: Congenital Heart Surgery Database
DoD: Department of Defense
HQH: High-quality Hospitals
ICD: International Classification of Diseases
MHS: Military Healthcare System
MDR: Military Health System Data Repository
O/E: Observed-to-Expected
OR: Odds Ratio
SES: Socioeconomic Status
STS: Society of Thoracic Surgeons
