# Supplementary material for: Does universal insurance influence disparities in high-quality hospital use for inpatient pediatric congenital heart defect care within the first year of diagnosis?
Source: BMC Health Serv Res. 2023 Jun 28;23:702. doi: 10.1186/s12913-023-09668-1 (PMC10303295; doi:10.1186/s12913-023-09668-1)
Supplement: Supplementary file 1 — Additional file 1. [file 12913_2023_9668_MOESM1_ESM.docx]

**Appendix A: Diagnosis Codes**

| **Diagnoses/procedures** | **ICD-10 codes** |
| --- | --- |
| **CHD diagnoses** |  |
| **Single ventricle** |  |
| Common ventricle | Q20.4 |
| Tricuspid atresia and stenosis, congenital (absence of tricuspid valve) | Q22.4 |
| Hypoplastic right heart syndrome | Q22.6 |
| Hypoplastic left heart syndrome | Q23.4 |
| **Moderate-complex CHD** |  |
| Common truncus | Q20.0 |
| Double outlet left or right ventricle | Q20.1, Q20.2 |
| Transposition of great arteries | Q20.3 |
| Corrected transposition of great vessels | Q20.5 |
| Isomerism atrial appendages | Q20.6 |
| Other congenital malformations of cardiac chambers and connections | Q20.8, Q20.9 |
| Endocardial cushion defect | Q21.2 |
| Tetralogy of Fallot | Q21.3 |
| Aorto-pulmonary septal defect | Q21.4 |
| Other/unspecified congenital malformations of cardiac septa | Q21.8, Q21.9 |
| Pulmonary valve atresia | Q22.0 |
| Pulmonary valve insufficiency | Q22.2 |
| Ebstein Anomaly | Q22.5 |
| Other congenital malformation of tricuspid valve | Q22.8, Q22.9 |
| Mitral stenosis and insufficiency | Q23.2, Q23.3 |
| Cor triatriatum | Q24.2 |
| Infundibular pulmonic stenosis | Q24.3 |
| Subaortic stenosis | Q24.4 |
| Coronary artery anomaly | Q24.5 |
| Congenital heart block | Q24.6 |
| Coarctation of aorta | Q25.1 |
| Atresia and stenosis of aorta | Q25.2, Q25.3 |
| Anomaly of aorta, unspecified | Q25.4 |
| Anomalies of aortic arch | Q25.4 |
| Aneurysm of sinus of Valsalva | Q25.4 |
| Pulmonary artery atresia | Q25.5 |
| Stenosis of pulmonary artery | Q25.6 |
| Other congenital malformations of pulmonary artery | Q25.7 |
| Other congenital malformations of great arteries | Q25.8, Q25.9 |
| Anomalies of great veins | Q26.0, Q26.1 |
| Total anomalous pulmonary venous connection | Q26.2 |
| Partial anomalous pulmonary venous connection | Q26.3, Q26.4 |
| **Diagnoses/procedures** | **ICD-10 codes** |
| Other/unspecified anomalies of great veins: | Q26.8, Q26.9 |
| Absence/ congenital stenosis of vena cava (inferior)(superior) |  |
| Anomaly of great veins, unspecified (Anomaly NOS of pulmonary | Q26.9 |
| veins, vena cava) |  |
| **Simple CHD** |  |
| Ventricular septal defect | Q21.0 |
| Atrial septal defect | Q21.1 |
| Pulmonary valve stenosis | Q22.1 |
| Other congenital valve malformation | Q22.3 |
| Aortic valve stenosis | Q23.0 |
| Aortic valve insufficiency | Q23.1 |
| Other congenital malformation of aortic and mitral valve | Q23.8, Q23.9 |
| Unspecified anomaly of heart | Q24.8, Q24.9 |
| Other/unspecified congenital malformations of circulatory system | Q28.8, Q28.9 |
| **Genetic Syndrome** |  |
| Down’s syndrome | Q90 |
| Noonan’s syndrome | Q87.1 |
| DiGeorge syndrome | D82.1 |
| Holt-Oram syndrome | Q87.2 |
| Turner syndrome | Q96 |
| Williams-(Beuren) syndrome | Q87.0 |
| **Comorbidities** |  |
| Acute bronchiolitis | J21.0, J21.8, J21.9 |
| Acute bronchitis | J20 |
| Acute upper and lower respiratory infection, unspecified | J06.9, J22 |
| Pneumonia | J18.9, J69.0 |
| Asthma, unspecified | J45.9 |
| Gastro-esophageal reflux without esophagitis | K21.9 |
| Gastro-esophageal reflux with esophagitis | K21.0 |
| Gastroentiris and colitis | A08.0, A08.4, A09.9, K52.9 |
| Malabsorption | K90.4 |
| Urinary tract infection | N39.0 |
| Acute renal failure, unspecified | N17.9 |
| Hernia | K40.2, K40.9, K42.9 |
| Failure to thrive | R62.8 |
| Fever, unspecified | R50.9 |
| Dehydration | E86.0 |
| Sepsis, unspecified | A41.9 |
